# Zebrafish GDNF and its co-receptor GFRα1 activate the human RET receptor and promote the survival of dopaminergic neurons *in vitro*

**DOI:** 10.1371/journal.pone.0176166

**Published:** 2017-05-03

**Authors:** Tuulia Saarenpää, Konstantin Kogan, Yulia Sidorova, Arun Kumar Mahato, Igor Tascón, Heidi Kaljunen, Liying Yu, Jukka Kallijärvi, Jaana Jurvansuu, Mart Saarma, Adrian Goldman

**Affiliations:** 1 Department of Biochemistry, Faculty of Biological and Environmental Sciences, University of Helsinki, Helsinki, Finland; 2 Institute of Biotechnology, University of Helsinki, Helsinki, Finland; 3 Astbury Centre for Structural Molecular Biology, Faculty of Biological Sciences, University of Leeds, Leeds, United Kingdom; Rutgers University, UNITED STATES

## Abstract

Glial cell line-derived neurotrophic factor (GDNF) is a ligand that activates, through co-receptor GDNF family receptor alpha-1 (GFRα1) and receptor tyrosine kinase “RET”, several signaling pathways crucial in the development and sustainment of multiple neuronal populations. We decided to study whether non-mammalian orthologs of these three proteins have conserved their function: can they activate the human counterparts? Using the baculovirus expression system, we expressed and purified *Danio rerio* RET, and its binding partners GFRα1 and GDNF, and *Drosophila melanogaster* RET and two isoforms of co-receptor GDNF receptor-like. Our results report high-level insect cell expression of post-translationally modified and dimerized zebrafish RET and its binding partners. We also found that zebrafish GFRα1 and GDNF are comparably active as mammalian cell-produced ones. We also report the first measurements of the affinity of the complex to RET in solution: at least for zebrafish, the *K*_d_ for GFRα1-GDNF binding RET is 5.9 μM. Surprisingly, we also found that zebrafish GDNF as well as zebrafish GFRα1 robustly activated human RET signaling and promoted the survival of cultured mouse dopaminergic neurons with comparable efficiency to mammalian GDNF, unlike *E*. *coli*-produced human proteins. These results contradict previous studies suggesting that mammalian GFRα1 and GDNF cannot bind and activate non-mammalian RET and *vice versa*.

## Introduction

Glial cell-line derived neurotrophic factor (GDNF), a secreted growth factor of the GDNF family ligands (GFLs) [1], is an important biomedical research target, especially since it has been shown to promote the survival of the midbrain dopaminergic neurons that degenerate in Parkinson’s disease [2]. GDNF and the other GFLs, neurturin, persephin, and artemin, each selectively pair with one of the four glycophosphatidylinositol- (GPI-) anchored co-receptors “GDNF family receptor α’s” (GFRαs) [3] to form a tripartite complex that signals through receptor tyrosine kinase RET [4, 5]. The proto-oncogene “Rearranged during Transfection” (RET) is also an important research target because its mutations are the cause of several pathologies including Hirschsprung’s disease and four different endocrine cancers (including multiple endocrine neoplasia 2A and medullary thyroid carcinoma), all due to a multitude of mutations occurring in the *RET* gene [6, 7, 8, 9]. RET was discovered three decades ago in mouse NIH3T3 cells transformed with human lymphoma DNA [10] and further characterization revealed it is a receptor tyrosine kinase. The overall architecture is as follows: the extracellular domain (ecd) contains four cadherin-like domains (CLD) with a calcium-binding site and a cysteine-rich domain; this is followed by an α-helical transmembrane domain and a cytosolic two-domain intracellular kinase [4, 5]. Upon formation of the RET-GFRα1-GDNF complex with a stoichiometry of 2:2:2 [11] the tyrosine kinases of the RET dimer autophosphorylate and these phosphorylated tyrosine residues serve as a binding platform for a number of adaptor proteins activating intracellular signaling pathways [3]. Ultimately, the activation of the various RET intracellular signaling pathways is important for promoting neuronal survival, neurite formation and branching, synapse formation, spinal motoneuron survival and also kidney development and spermatogenesis [12, 13].

RET is highly evolutionarily conserved and orthologs have been found in several classes of organisms including amphibians, fish and even insects [14, 15, 16]. GDNF and GFRαs are found in all vertebrates and the sequence identity amongst them is also high. The overall amino acid sequence identities of the vertebrate proteins are 50% and above, but the RET kinase domain orthologs have a sequence identity of up to 80%. Despite the high conservation of the proteins between species, previous research has failed to show any interaction between mammalian RET and non-mammalian GFRα1 or GDNF, or *vice versa*.

There is as yet no x-ray structure of a RET tripartite complex, just of parts of it: GDNF [17], GDNF-GFRα1 [18], artemin-GFRα3 [19], the intracellular domain of RET [20], and the first two CLDs [21]. Kjær *et*. *al*. proposed that mammalian RET has unique sequence features that make it more susceptible to misfolding than non-mammalian RET orthologs [21], explaining why it has been hard to express and purify. In addition, previous experiments have signally failed to find interactions between mammalian GFRαs and ligands to any non-mammalian RET receptor. On the contrary, previous work shows that mammalian GFRα1/GDNF detects neither *Xenopus laevis* RET^ecd^ [22] nor *D*. *melanogaster* RET^ecd^ [23]. Kjær *et al*. thus further proposed that the unique features of mammalian RET explain this lack of cross-reactivity [21].

We wanted to investigate if the distinction between mammalian and non-mammalian RET was as stark as had been proposed. We have thus investigated the expression and functionality of various non-mammalian proteins of the RET complex using the baculovirus expression system (BEVS) in insect cells. We expressed and purified zebrafish (*Danio rerio*) RET, GFRα1, GDNF and fruit fly (*Drosophila melanogaster*) RET and two isoforms (A and Ab) of GDNF family receptor-like (GFRL) to study whether they interact with mammalian RET proteins and whether they promote the survival of mammalian neurons, as this might provide insights into the structural requirements of the signaling complex.

## Materials and methods

### Animal tissue

Zebrafish tissue (a kind gift from Prof. Pertti Panula) was collected by the donating laboratory by their ethical permit. The zebrafish Turku line that has been maintained in the laboratory for over a decade and used in several studies was used ([24, 25]). The permits for the experiments were obtained from the Office of the Regional Government of Southern Finland in agreement with the ethical guidelines of the European convention. Mice used in this study were anesthetized using CO_2_ prior to decapitation. Animal experiments was performed according to guidelines provided by EU legislation, Finnish legislation and have been proved by National Animal Experiment Board of Finland and use committee (permit no. KEK15-022).

### Cloning and plasmid constructs

*dRET*^*ecd*^ (residues 30–696; UniProt accession number Q7KT06), and *dGFRLA* and *dGFRLAb* [26] cDNA were cloned into the pK503.9 vector (pFastbac1 derivative) [27] modified to have eight histidine residues and a thrombin cleavage site, herein pK503.9-His_8_-Thr. *dRET*^CLD1-CLD4^ lacking the cysteine-rich domain (residues 30–520) was cloned into the original pK503.9 vector (Flag-tag only). Zebrafish tissue was collected by the donating laboratory. In brief, RNA was extracted from frozen 2 days post-fertilization (dpf) and 7-dpf zebrafish tissue with Tripure reagent (Roche) according to the manufacturer’s protocol. Total RNA was reverse transcribed with Superscript II (Thermo Fisher Scientific). *zGFRα1* (residues 31–351; UniProt accession number Q98TT9) lacking the GPI-anchor was cloned into pK503.9-His_8_-Thr and mature *zGDNF* (residues 90–236; UniProt accession number Q98TU0) was cloned into pK503.9-His_8_-Thr (His-tagged zGDNF) and the original pK503.9 vector (zGDNF). *zRET*^*ecd*^ (residues 22–626; UniProt accession number A8E7C6) was cloned into the pK503.9-His_8_-Thr from zRET-CS2-MT+ [15] (a kind gift from Prof. David Grunwald). Mature *hGDNF* (residues 142–275; UniProt accession number P905) was cloned into the pK503.9-His_8_-Thr vector from original plasmid described in [28], herein referred to as hGDNF^BV^.

### Cell culture and transfection

Sf9 and Hi5 cells (Thermo Fisher Scientific) were grown and maintained in HyClone SFX-insect medium (Thermo Fisher Scientific) at a density of 1.5–2.5 million cells/ml in suspension, shaking on an orbital shaker at 90–120 rpm at 27°C until transfection. Recombinant baculovirus DNA (bacmid) was prepared according to the Bac-to-Bac manual (Thermo Fisher Scientific) and zRET^ecd^ virus was prepared as described elsewhere [29]. Virus was amplified from passage 0 (V0) to passage 2 (V2) in Sf9 cells. For protein expression, Hi5 cells were infected with V2 virus and media was harvested after 72 hours of shaking and incubation at 27°C.

### Purification of recombinant proteins

dRET^ecd^, dGFRLA, dGFRLAb, zRET^ecd^, and zGFRα1, hGDNF^BV^, and His-tagged zGDNF were all purified using nickel affinity chromatography and size exclusion chromatography (SEC) using low-pressure flow chromatography. Media was harvested by centrifugation at 1500 g for 20 minutes at 4°C and filtration with a 0.8 μm membrane (Whatmann). For cultures over 1L we used a Pellicon concentrator (Millipore EMD) to concentrate and exchange the media to binding buffer (50 mM Tris-HCl pH 8, 150 mM NaCl, 20 mM imidazole pH 8). The retentate was loaded onto a 1 mL HisTrap HP column (GE Healthcare) equilibrated with binding buffer and the His-tagged proteins were eluted using a linear gradient of elution buffer (50 mM Tris-HCl pH 8, 150 mM NaCl, 20 to 500 mM imidazole pH 8). 1 mM CaCl_2_ was always added to buffers for dRET^ecd^ and zRET^ecd^ purifications. Cell culture media for expressing zGDNF was harvested as above and it was loaded onto a 1 mL HiTrap Heparin HP column (GE Healthcare) equilibrated with binding buffer (20 mM HEPES pH 8, 100 mM) and eluted with a high concentration salt gradient (100 mM to 1 M NaCl). All gradient elutions were performed on an Äkta purifier (GE Healthcare) by collecting peak fractions observed by UV 280 nm on the chromatogram and analyzing the samples by SDS-PAGE and Western blotting with anti-Flag M1 antibody (1:5000, F3040, Sigma Aldrich) and goat anti-mouse antibody (1:5000, sc-2031, Santa Cruz Biotechnology, Inc.) according to the manufacturer’s protocols. Positive fractions were then pooled and dialyzed against gel filtration buffer (20 mM HEPES pH 8, 150 mM NaCl). The Flag and His-tag were cleaved off with thrombin (10 units per 1 mg of protein, Sigma Aldrich) at this stage according to the manufacturer’s instructions in all experiments except where we specifically mention that the His-tag was present. The sample volume was reduced to 0.5 mL for SEC using Amicon centrifuge concentrators (Millipore EMD) with the appropriate molecular weigh cut-off and loaded onto the Superdex 200 GL 10/300 column (GE Healthcare) equilibrated with gel filtration buffer. Deglycosylation experiments were done with PNGase F enzyme (Santa Cruz Biotechnology, Inc.) according to the manufacturer’s protocol.

### Binding experiments

dRET^CLD1–4^ and dGFRLA binding experiments were performed by co-expressing the proteins in Hi5 cells. The cells were infected with molar equivalent ratios of V2 viruses and incubated for 72 hours. Co-expressions were performed with and without 1 mM CaCl_2_. The binding of dRET^ecd^ to dGFRLAb was tested by immobilizing purified His-tagged dGFRLAb to His SpinTrap columns (GE Healthcare), incubating with dRET^ecd^ (without its His-tag) for 15 minutes, washing twice with 20 mM wash buffer (as above), and eluting with 250 mM imidazole elution buffer. Samples of each fraction were collected and analyzed by sodium dodecyl sulfate polyacrylamide gel electrophoresis (SDS-PAGE).

The interaction between zRET^ecd^, His-tagged zGFRα1, and zGDNF was determined by biolayer interferometry using the BLItz system (ForteBio Inc.) Ni-NTA biosensor tips (ForteBio Inc.) were pre-wetted with gel filtration buffer (with 1 mM CaCl_2_) for 10 minutes. 4 μl of His-tagged zGFRα1 (58 μM) was immobilized onto the biosensor tip. For the tripartite complex, six concentrations of pre-mixed zRET^ecd^ and zGDNF were used for detection: 0.3, 1.4, 5.6, 11, 22, and 44 μM. Measurements were done according to the manufacturer’s instructions. The raw data were exported into GraphPad Prism 6, plateau binding values fit as Scatchard plots to obtain *K*_d_s. Measurements were done in triplicate.

### RET phosphorylation assays

To assess human RET (hRET) phosphorylation, MG87RET cells transiently transfected with human GFR0α1 (hGFRα1) were treated with a final concentration of 0.27, 1.35, and 6.75 nM of zGDNF, hGDNF^BV^ human GDNF produced in mammalian cells (hGDNF^IC^) (Icosagen), and in *E*. *coli* (hGDNF^Ec^) (Prospec). Cells were washed once with ice-cold 1x phosphate-buffered saline (PBS) containing 1 mM Na_3_VO_4_ and lysed with 500 μl per well of RIPA-modified buffer [50 mM Tris-HCl pH 7.4, 150 mM NaCl, 1 mM EDTA, 1% NP-40, 1% TritonX-100 (TX-100, Sigma Aldrich), 10% glycerol, EDTA-free protease inhibitor cocktail (Roche), 1 mM Na_3_VO_4_, 2.5 mg/ml sodium deoxycholate, 1 mM phenylmethanesulfonyl fluoride (PMSF)]. To immunoprecipitate hRET, the lysates were incubated with 2 μg/ml anti-RET C-20 antibody (Santa Cruz Biotechnology, Inc.; sc-1290) and Dyna Protein G beads (Life Technologies AS) overnight at 4°C. The resulting pellets were washed three times with 1x Tris-buffered saline (TBS) supplemented with 1% TX-100. Immunoprecipitated proteins were resolved on 7.5% SDS-PAGE and then transferred onto a nitrocellulose membrane. Membranes were blocked with blocking solution (3% non-fat milk in TBS-T (1xTBS with 0.1% Tween)) for one hour and probed with anti-phosphotyrosine antibody (1:500; Merck Millipore; 05–321) in blocking solution overnight at 4°C. Membranes were washed three times for 15 minutes in TBS-T and incubated with HRP-conjugated polyclonal goat anti-mouse secondary antibody (1:3000; Dako; P044701-2) in blocking solution. Membranes were washed four times for 15 minutes with TBS-T. Bound antibodies were visualized with ECL reagent (Pierce) using a LAS3000 Luminescent Image Analyzer (Fuji-Film). The loading controls were assessed by reprobing the same membrane with anti-RET C-20 antibody (1:500, Santa Cruz Biotechnology, Inc.; sc-1290) for one hour at room temperature. Membranes were washed, incubated with HRP-conjugated polyclonal rabbit anti-goat secondary antibody (1:500; Dako; P04491-2) in blocking solution and visualized according to above-mentioned procedure. The phospho-RET ELISA assays were done with the same concentrations of GDNFs (zGDNF, hGDNF^BV^, GDNF^IC^) as mentioned earlier according to methods described elsewhere [18]. All measurements were done in triplicate.

### Luciferase assays

To monitor the activation of the MAPK/ERK signaling cascade in MG87 fibroblasts expressing either hRET only or hRET and hGFRα1, we used a luciferase reporter as described elsewhere [28]. One day before the assay, cells (MG87RET expressing hGFRα1 to test zGDNF and MG87RET to test testing both zGDNF and zGFRα1) were plated in 96-well plates at a density of 2 x 10^5^ cells/well in Dulbecco’s Modified Eagle Medium (DMEM) Thermo Fisher Scientific) with 10% fetal bovine serum (FBS), and 100 μg/ml normocin. The next day we applied the purified proteins. We used a final concentration of 0.27, 0.68, 1.35, 2.7, 6.75 and 13.5 nM of zGDNF, and hGDNF^Ec^, hGDNF^BV^, and hGDNF^IC^ as positive controls and DMEM as a negative control. To assess the activity of zGFRα1 with zGDNF, the final concentrations of pre-incubated zGDNF/zGFRα1 were 0.14/0.5, 0.27/1, 0.68/2.7, 2.7/10.8, 6.75/27, and 13.5/54 nM. We used media, zGDNF alone at 0.14 and 0.27 nM, and zGFRα1 alone at 0.5 and 1 nM as negative controls. After the addition of protein, cells were cultivated for 24 hours to express luciferase. Luciferase activity was determined by lysing the cells in Passive Lysis Buffer (Promega), adding 20 μl Luciferase Assay Reagent (Promega) in 10μl of lysates and luminescence on a Microbeta 2 plate reader (Perkin Elmer). We also assessed whether presence of an N-terminal His-tag on both zGDNF and zGFRα1 had negative impact on the activity of hRET using the same methods above individually with different concentrations of either His-tagged zGDNF or zGFRα1. Luciferase assays in MG87TrkB cells expressing luciferase reporter were performed as described in [28]. All measurements were done in triplicate.

### Assaying survival of cultured neurons

E13.5 pregnant mice were sacrificed and embryos were collected in a sterile Petri dish containing DMEM. The ventral midbrain was isolated and midbrain floors were dissected under a dissection microscope (Olympus SZX10 Stereo Microscope). Isolated tissues were cut into small pieces with syringes, collected in Eppendorf tubes, and washed three times with Calcium and Magnesium-free Hank’s Balanced Salt Solution (HBSS) (Gibco, Life Technologies; 14170161) and incubated in 5 mg/ml trypsin solution in HBSS for 20 minutes at 37°C. Enzymatic digestion was blocked by fetal bovine serum (FBS). DNase I (10 mg/ml) was added to the sample to degrade released genomic DNA and to reduce the viscosity of the solution. Tissues were mechanically dissociated using fire-polished Pasteur pipettes to obtain a single cell suspension. The dissociated cells were centrifuged and washed three times with cell culture media [Dulbecco’s MEM/Nut mix F12 (Invitrogen/Gibco; 21331–020), 1xN_2_ serum supplement (Invitrogen/ Gibco; 17502–048), 33mM D-Glucose (Sigma; G-8769), 0.5 mM L-Glutamine (Invitrogen/Gibco; 25030–032), and 100 μg/ml Primocin (Invivo Gen)]. Cells (30,000 cells/well) were plated on a 96-well plate pre-coated with Poly-L-ornithine (Sigma Aldrich), as described in more detail elsewhere [30]. The plates were incubated at 37°C for 5 days. Different concentrations of purified zGDNF and hGDNF^IC^ (0.027, 0.14, 0.27, 0.4, 0.81, and 2.7 nM) were applied within one hour of plating. The culture media was partially replaced after two days. Cells were cultured for five days and then fixed with 4% paraformaldehyde (PFA) in 1x PBS for 20 minutes. Dopaminergic neurons were visualized with Anti-Tyrosine Hydroxylase antibody (1:500; Millipore EMD; MAB318) and Alexa Fluor 647-conjugated secondary antibody (1:500; Thermo Fisher Scientific; A-31571). Nuclear staining was performed using 0.2 μg/ml DAPI (4’, 6-diamidino-2-phenylindole) in 1x PBS for 10 minutes at room temperature. After washing cells were left in 1x PBS and imaged using a CellInsight CX5 (Life Technologies). The number of TH-positive cells was quantified using cell Profiler image analysis software (BROAD institute). 0- to 2-day postnatal rat superior cervical ganglia (SCG) neurons were cultured in the presence of 0–2.7 nM zGDNF or 1.35 nM of hGDNF^Ec^ (PeproTech Ltd.) as a positive control for five days. Viable neurons were counted daily and expressed as percentage of hGDNF^Ec^ present neurons. Results are presented as mean ± SEM of three independent experiments.

### Statistical analysis

Statistical analyses were performed using GraphPad Prism 6. To detect statistically significant differences we used one-way ANOVA and the Dunnett (to compare results to control) or Tukey (to compare results to each other) post-hoc tests.

## Results

### Insect cells express correctly-folded Ret complex signaling proteins

**Fig 1:**
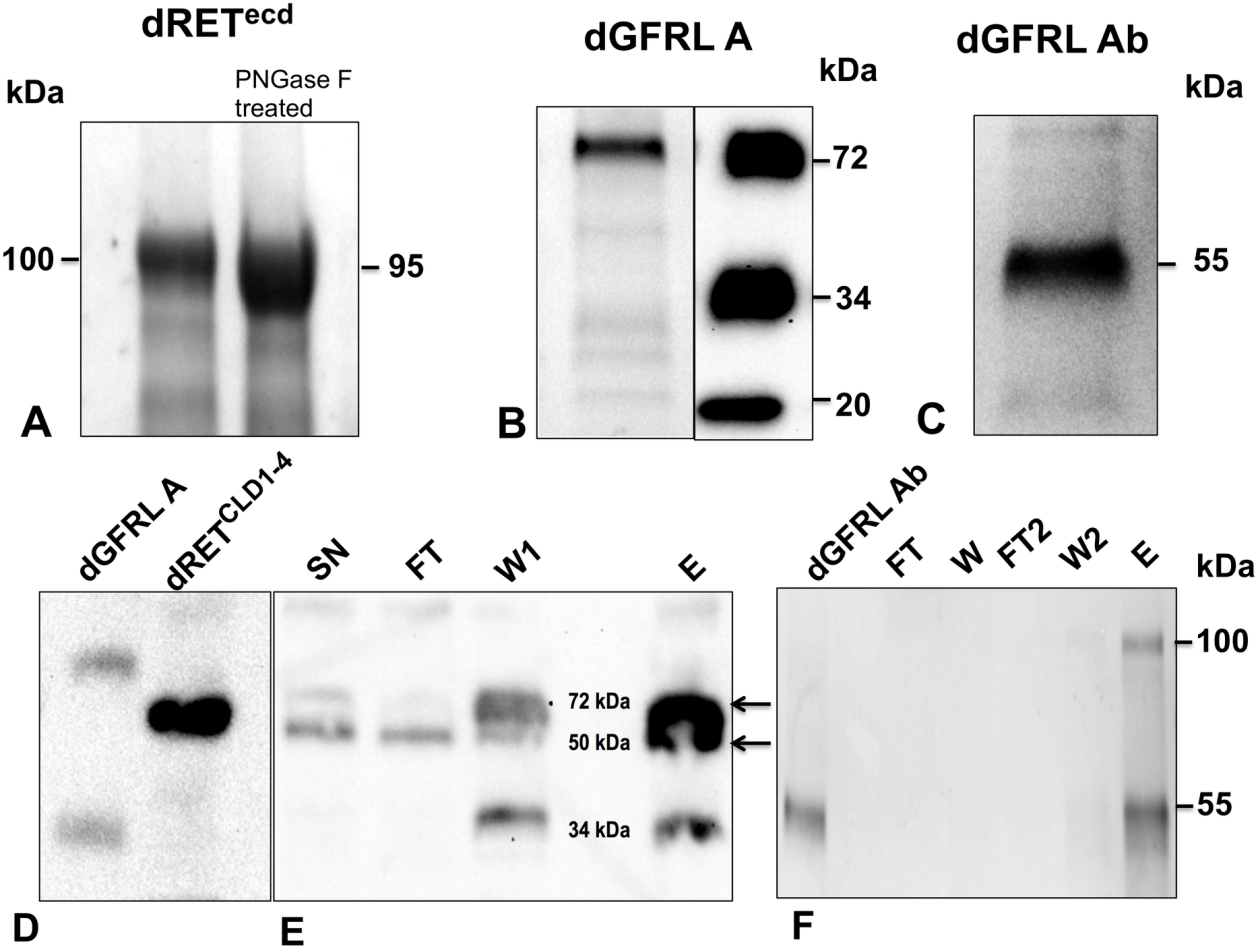
The BEVS successfully expressed secreted and folded fruit fly RET and GFR-like A and Ab proteins. (A) purified dRET^ecd^ on a Coomassie brilliant blue stained gel reveals that native dRET^ecd^ (-) migrated higher than PNGase F treated dRET^ecd^ (+). (B) (left panel) purified dGFRLA on a Coomassie brilliant blue stained gel reveals that it migrated at a molecular weight of 72 kDa (right panel) and anti-Flag Western blotting identified possible proteolytic cleavage fragments of dGFRLA at 34 kDa and 20 kDa. (C) purified dGFRLAb migrated on a Coomassie brilliant blue stained gel at a molecular weight of 55 kDa. (D) Anti-Flag Western blotting showing dGFRLA and dRET^CLD1-4^ controls and (E), pull-down of co-transfected dGFRLA and dRET^CLD1-4^ where most of the dRET^CLD1-4^ was present in the elution. (F), Coomassie stained gel of a pull-down assay using His-tagged dGFRLAb and untagged dRET^ecd^, where dRET^ecd^ was only seen in the elution. *SN*, supernatant; *FT*, flow-through; *W*, wash; *E*, elution.

We expressed recombinant dRET^ecd^, dRET^CLD1-4^, the two isoforms of dGFRL (dGFRLA, and dGFRLAb) using standard methods for baculovirus/insect cell expression. All the proteins were secreted into the media, from which we purified them using affinity chromatography and size exclusion chromatography (SEC). Samples of the protein purification eluates were analyzed with SDS-PAGE, revealing that native dRET^ecd^ migrated at 100 kDa (Fig 1*A*), although the calculated molecular weight of the protein is 78 kDa. dRET^ecd^ glycosylation contributes to the higher than expected MW of the purified protein, as it has six predicted N-glycosylation sites. Treatment with PNGase F resulted in a band migrating at 90 kDa (Fig 1*A*), but the SDS-PAGE showed that the protein was not fully glycosylated. The purified dGFRL proteins also migrated as a single band, isoform A at 72 kDa (Fig 1*B*) and isoform Ab at a single band at 55 kDa (Fig 1*C*). Pull-down assays of dRET^CLD1-4^ with dGFRLA (Fig 1*D*) and dGFRLAb (Fig 1*E*) showed that the untagged dRET was present in the elution but not in the flow-through or wash steps. dRET thus binds dGFRLs.

**Fig 2:**
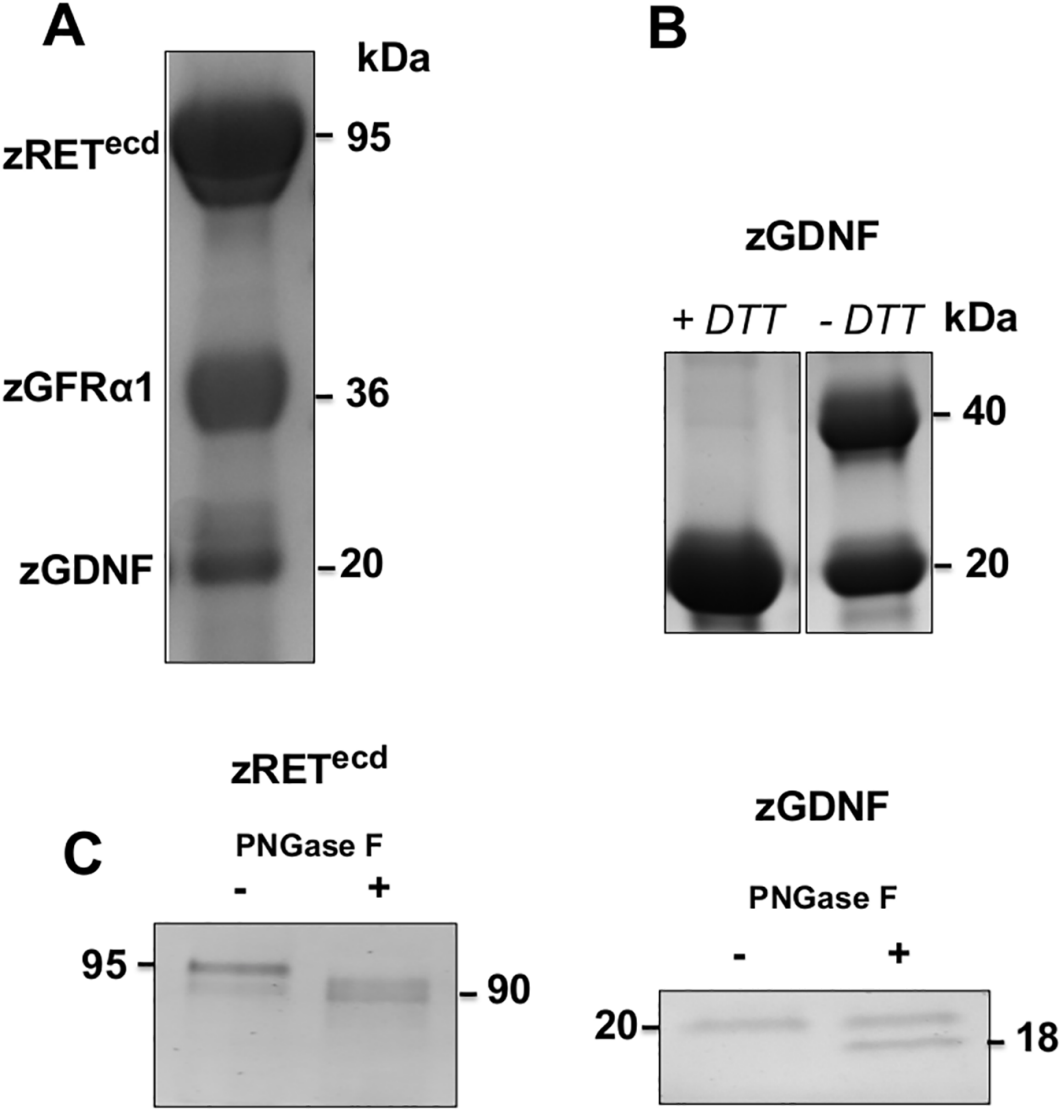
The BEVS successfully expressed secreted and correctly folded zebrafish RET^ecd^, GFRα1 and GDNF. (A) Purified zRET^ecd^, zGFRα1 and zGDNF on a Coomassie stained gel at corresponding molecular weights of 95 kDa, 37 kDa and 20 kDa. (B) zGDNF monomer and dimer on a Coomassie stained gel, monomer seen in 1 mM DTT (+) treated sample and dimer seen without (-) DTT. (C) both zRET^ecd^ and zGDNF were sensitive to PNGase F treatment: zRET shifted from 95 kDa to 90 kDa and zGDNF from 20 kDa to 18 kDa on Coomassie stained gel.

**Fig 3:**
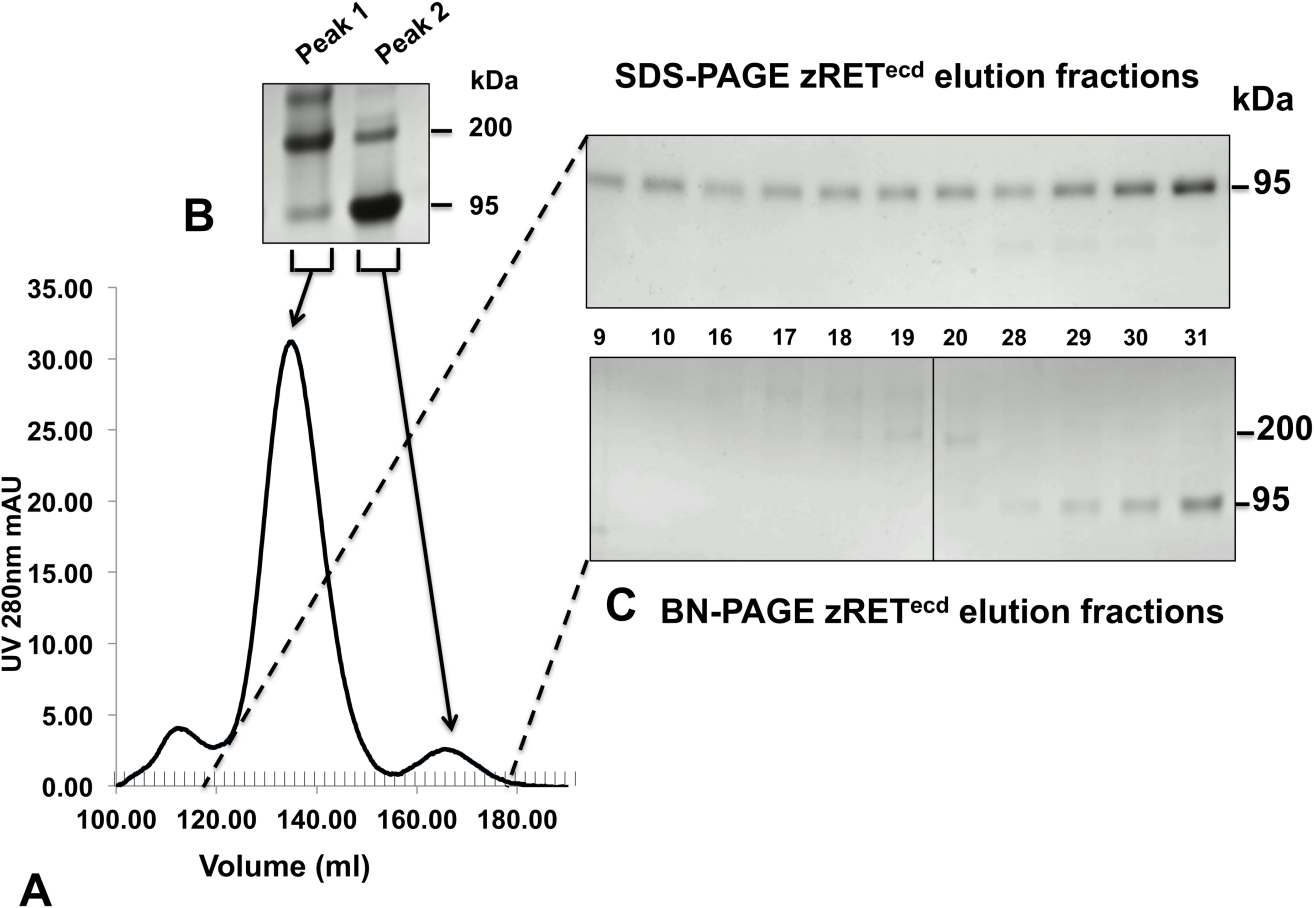
Purified zRET^ecd^ exists as a dimer and monomer in solution. (A) SEC separated affinity chromatography purified zRET^ecd^ as two populations of protein eluting as two peaks (peak 1 on the left and peak 2 on the right) as seen from the chromatogram. (B) Pooled and concentrated fractions of peak 1 and peak 2 run on BN-PAGE. The dimer eluted from 120 ml to 155 ml, migrating at 200 kDa, and monomer eluted at 156 ml to 180 ml, migrating at 95 kDa in BN-PAGE. (C) Elution fraction samples were collected from both peak 1 and peak 2 and the same fractions (numbered between the images) run on SDS-PAGE and BN-PAGE. For both peaks, a single 95 kDa band was seen in SDS-PAGE and two bands were seen in BN-PAGE.

We also expressed and purified recombinant zRET^ecd^, zGFRα1, and zGDNF in the same way. All zebrafish protein samples migrated as single bands under reducing conditions on SDS-PAGE (Fig 2*A*), all at approximately their predicted molecular weights with the exception of zRET^ecd^, due to post-translational glycosylation. Comparing zGDNF samples incubated in reducing and non-reducing conditions showed that native zGDNF ran as two bands and reduced zGDNF as one (Fig 2*B*). Recombinant zGDNF appears to exist in solution as a mixture of dimer and monomer. Both zRET^ecd^ and zGDNF were sensitive to PNGase F treatment (Fig 2*C*), consistent with the predicted N-glycosylation sites on both proteins. Size exclusion chromatograms of zRET^ecd^ purifications consistently showed two peaks in the chromatogram indicating a heterogeneous population of protein (Fig 3A). Analyzing sample individual fractions and concentrated pools of each peak on both SDS-PAGE and BN-PAGE indicate that the two protein populations are presumably a dimer and monomer of zRET (Fig 3*B* and 3*C*).

**Fig 4:**
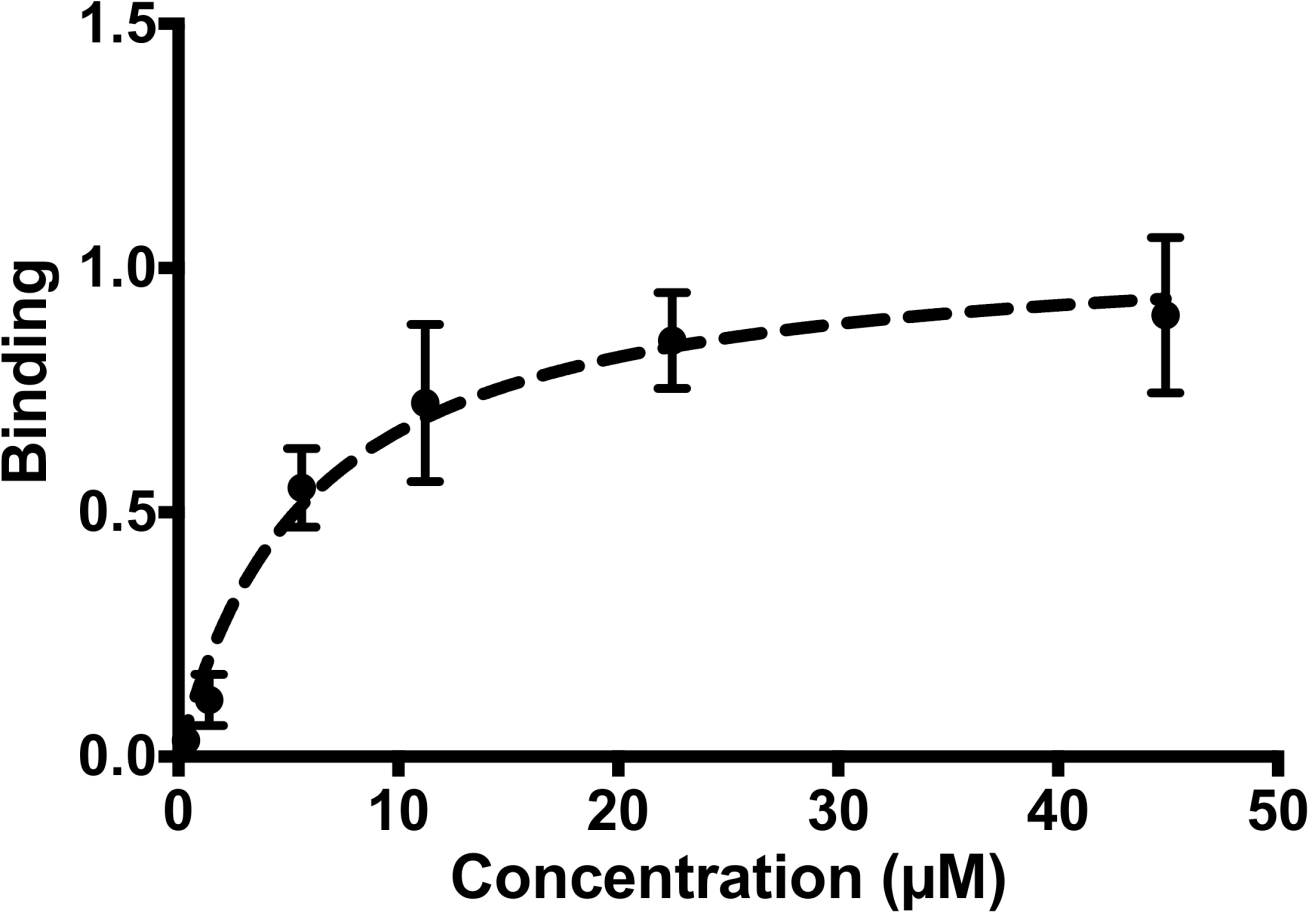
Biolayer inferometry (BLItz) of zebrafish proteins. Scatchard plot showing the binding of zRET^ecd^ and zGDNF to pre-incubated zGFRα1 using BLItz. The x-axis shows the concentration of zGDNF and zRET^ecd^. The dashed line indicates the nonlinear curve fit plotted by GraphPad Prism 6 for single-site binding.

We used Scatchard plots to analyze the binding affinities of the zebrafish proteins for each other when zGFRα1 was attached to the biosensor. zRET^ecd^ bound the zGFRα1-zGDNF complex with a *K*_d_ of 5.9 μM ± 1.5 (Fig 4). As expected, zRET did not bind zGDNF in the absence of zGFRα1 (data not shown).

**Fig 5:**
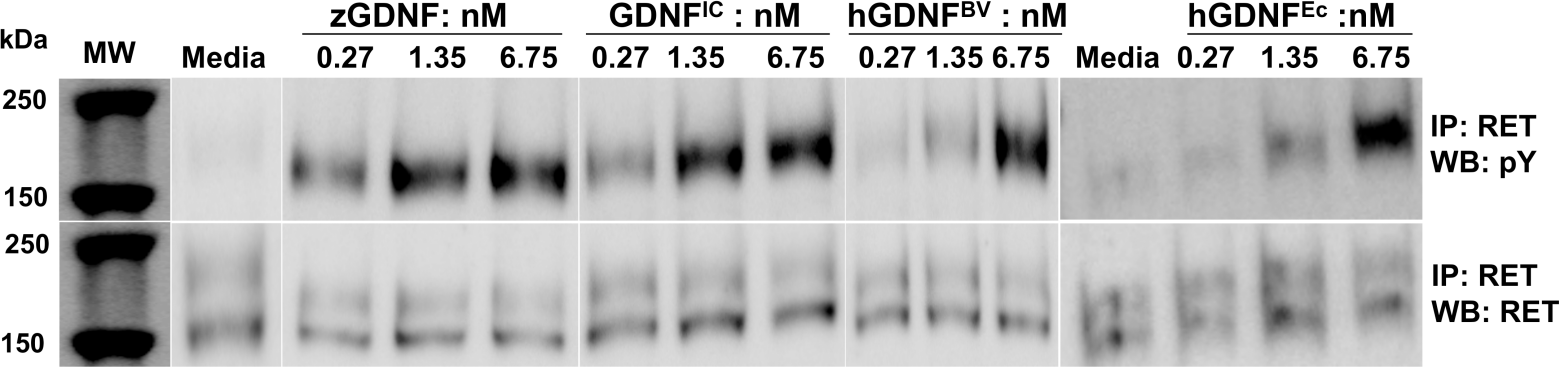
zGDNF activates hRET phosphorylation as shown by anti-phospho-RET western blotting. The anti-phosphotyrosine (pY) Western blotting reflects the level of phosphorylated tyrosine residues in RET (upper panels); anti-RET western blotting (lower panels) demonstrates equal loading in different samples. hGDNF^IC^, hGDNF^BV^ and hGDNF^Ec^ produced hGDNF were used as controls at a range of concentrations stated in nM and media as a negative control.

We next assessed whether zGDNF or dGFRL were able to activate human RET (hRET) phosphorylation in a relevant cellular model, the MG87RET mouse fibroblast cell line [31] transiently transfected with hGFRα1. Immunoblotting against phosphorylated hRET showed that zGDNF clearly stimulated autophosphorylation at concentrations ranging from 0.27 to 6.75 nM at a similar efficiency as the various controls including human GDNF produced in insect cell/baculovirus (hGDNF^BV^), in mammalian cells (hGDNF^IC^), and in *E*. *coli* (hGDNF^Ec^) (Fig 5). To confirm this, we repeated the experiment using a luminescence-based ELISA assay, but this time comparing only GDNF produced in eukaryotic cells. zGDNF and hGDNF showed a significantly higher level of activation than hGDNF^BV^ at all tested concentrations, and zGDNF showed statistically higher activation compared to hGDNF^IC^ at concentrations of 0.3 and 1.5 nM (S1 Fig). These results clearly indicate that recombinant zGDNF activates the human RET / GFRα1 complex.

**Fig 6:**
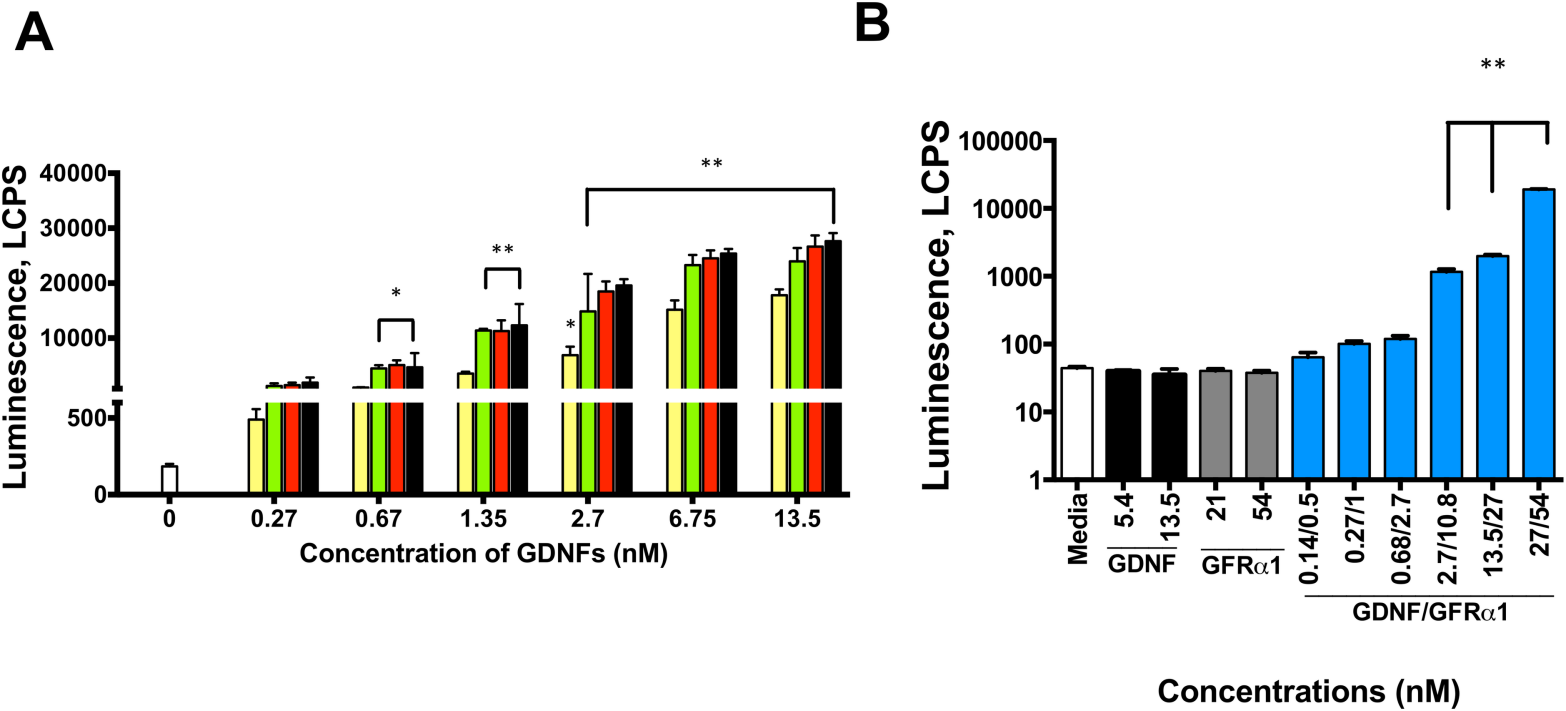
Zebrafish GDNF alone and with zGFRα1 potently activated the human RET MAPK signaling. (A) luciferase activity readouts comparing different concentrations of zGDNF, using hGDNF^BV^, hGDNF^Ec^ and hGDNF^IC^ as positive controls. Yellow bar: hGDNF^Ec^, green bar: hGDNF^BV^; red bar: hGDNF^IC^; black bar: zGDNF. (B) luciferase activity readouts comparing zGDNF and zGFRα1 activation at different concentrations on the MG87 cell-line expressing RET only. Media, zGDNF and His-tagged zGFRα1 individually were used as negative controls. N = 3–4 repeats per experiment; statistically different from control *p< 0.01; ** *p*<0.0001. Error bars indicate SEM.

We then assessed whether zGDNF signaling of the hRET complex leads to the activation of mitogen-activated protein kinases/extracellular signal-regulated kinases (MAPK/ERK) pathway using a luminescence-based MAPK activation reporter gene assay [28] as in principle the previous results could have been due to adventitious phosphorylation of the wrong tyrosine. Our results showed that zGDNF in concentrations ranging from 0.67 to 13.5 nM stimulated RET-dependent MAPK/ERK with comparable potency to hGDNF^IC^, hGDNF^BV^ and hGDNF^Ec^ (Fig 6*A*). zGDNF exhibited similar signaling activity compared to hGDNF^IC^ and hGDNF^BV^ and significantly more than hGDNF^Ec^ at concentrations from 0.67 to 13.5 nM using the Tukey comparison posthoc test. We also found that an N-terminal Flag and 8-His tag on the recombinant zGDNF significantly inhibited signaling through hRET (S2*A Fig*).

Since zGDNF apparently activated hRET / hGFRα1 normally, we decided to investigate whether *hGFR*α*1* was required for this signaling or whether *zGFR*α*1* would be as effective at activating hRET (*i*.*e*. is it that hRET interacts with hGFRα1, and zGDNF then interacts with hGFRα1 through the conserved interface, or the zebrafish system sufficient). We stimulated MG87RET cells that express only hRET (no hGFRα1), again using the luciferase assay to monitor MAPK/ERK pathway activation with both zGDNF and zGFRα1. To exclude possible contamination in the samples that could activate hRET independently of zGDNF and zGFRα1, we stimulated hRET with zGDNF and zGFRα1 alone. zGDNF together with zGFRα1 significantly activated hRET signaling from a concentration of 2.7 nM zGDNF and 10.8 nM zGFRα1 up to the highest concentration tested, but there was no signal if only one of the two proteins was used (Fig 6*B*). While investigating the activity of zGDNF, we noticed that placement of an N-terminal His-tag had a negative impact on signaling (S2*A* Fig) (see above). Thus, we also tested whether the N-terminal Flag and 8-His tag had any effect on zGFRα1 function using the same system. There was no significant difference in the activity of zGFRα1 with or without this tag (S2*B* Fig).

Due to the fact that we observed binding of dGFRL to dRET (Fig 1*D* and 1*E*), we investigated whether dGFRL Ab potentially elicited phospholyation of hRET using the luciferase assay in the same manner as for the zebrafish proteins. No GDNF ortholog for fruit fly has been discovered yet so we used zGDNF and hGDNF for the experiments. We observed no significant increase in the activity of hRET when stimulated with dGFRL Ab (data not shown).

Since GDNF acts as a ligand for several receptors [32], we used a reporter cell line in which hRET was replaced by the Tropomycin receptor kinase B (TrkB), as described previously [28], to exclude the possibility of RET-independent activation of luciferase by zGFRα1 and zGDNF. Neither human or zebrafish GDNF (S3*A* Fig) nor zGDNF/zGFRα1 and hGDNF/zGFRα1 (S3*B* Fig) activated luciferase expression *via* MG87TrkB in reporter cells. These results show that in addition to inability to activate TrkB, zGDNF and zGFRα1 also do not activate the other receptors in the MG87 cells.

### Zebrafish GDNF supports the survival of cultured neurons similarly to human GDNF

**Fig 7:**
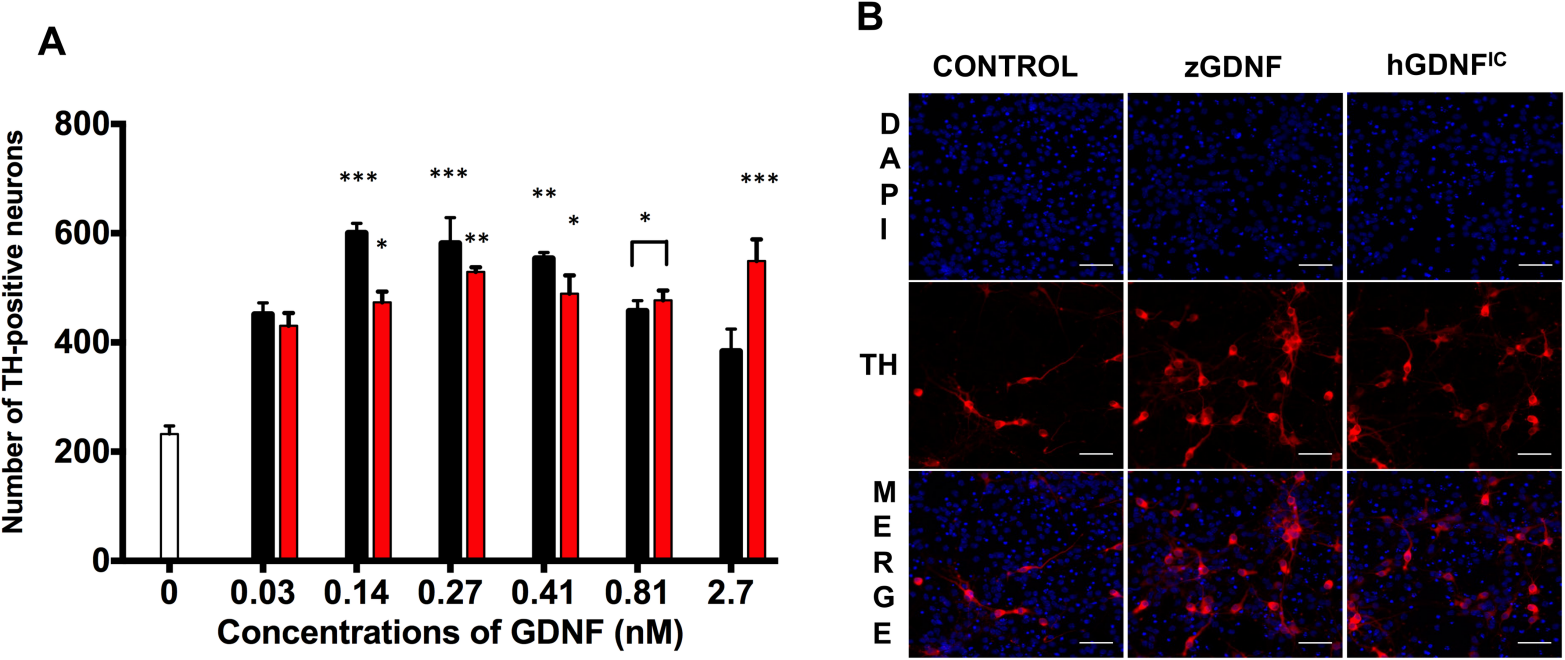
zGDNF supports the survival of cultured mammalian dopaminergic neurons. (A) the counted number of embryonic dopaminergic neurons in the presence of zGDNF and hGDNF^IC^ versus negative control (media), (B) immunofluorescent staining of midbrain culture on 5DIV with a marker for DA neurons: TH (red) and nuclear dye DAPI (blue) comparing 0.27 nM of hGDNF^IC^, zGDNF and no GFL (control). Black bars: zGDNF; red bars: hGDNF^IC^; white bar: negative control. N = 4 indiviaul experiments per condition; statistically different from control ***p< 0.0007, **p< 0.003, *p<0.03.

We next wanted to see whether zGDNF could support the survival of neurons as well as human GDNF does. We investigated whether zGDNF promoted the survival of mouse dopaminergic (DA) neurons in culture, using hGDNF^IC^ as a positive control. DA neurons were visualized using antibodies against tyrosine hydroxylase (TH), the key enzyme of dopamine synthesis. zGDNF was indeed able to promote the survival of DA neurons in a similar manner to hGDNF at concentrations from 0.14 to 0.41 nM, but at the highest concentrations (0.81 and 2.7 nM GFL) the survival of dopaminergic neurons decreased (Fig 7*A*). Examination of the immunofluorescent images indicates that in the cultures of DA neurons treated with 0.27 nM GFL a comparable amount of TH positive cells (red) were observed in the wells treated with zGDNF compared with hGDNF^IC^ (Fig 7*B*).

We also cultured another GDNF-responsive neuronal population, rat superior cervical ganglion (SCG) neurons in the absence or presence of either 1.4 or 2.7 nM zGDNF, or 1.4 nM hGDNF^Ec^ for five days. In the absence of any trophic support, there was a significant decrease in the number of live neurons on day 5, but both hGDNF^Ec^ and zGDNF promoted the survival of the cultured neurons with comparable efficiency (S4 Fig). Overall, our zGDNF produced with the BEVS appears to promote survival of several types of mammalian neurons as effectively as commercial human GDNF.

### Sequence alignment analyses

We then analyzed the various RET protein sequences to try and understand why zebrafish GDNF / GFRα1 was able to activate human RET and other non-mammalian proteins (*e*.*g*. frog and fruit fly) were not [23, 22]. As expected, fruit fly proteins only had 10–20% identities to any of the others (Table 1). Sequence alignments of dGFRL Ab, zGFRα1 and hGFRα1 show two 100 amino acid extensions in the sequence compared to the former. Comparison of homology modelled dGFRL Ab and the known 3D structure of rat GFRα1 showed that the structures of domain 2 and 3 are conserved, but the long amino acid extensions form protrusions (S5 Fig).

**Table 1:** Sequence identity comparisons of the amino acid sequences of human, zebrafish, and frog GDNF.

	Human GDNF	Zebrafish GDNF	Frog GDNF
**Human GDNF**	100%	48.5%	56.8%
**Zebrafish GDNF**	48.5%	100%	48.1%
**Frog GDNF**	56.8%	48.1%	100%

Frog (*Xenopus laevis*) RET proteins have higher sequence identity to human RET proteins than zebrafish proteins do (Tables 1–3): for instance frog GDNF is 57% identical to hGDNF, while zGDNF is 49% identical (Table 1). Surprisingly, however, zGDNF signals through hRET, while frog GDNF [22] does not. Why is this so?

**Table 2:** Sequence identity comparisons of the amino acid sequences of human, zebrafish, frog GFRα1 and fruit fly GFRL.

	Human GFRα1	Zebrafish GFRα1	Frog GFRα1	Fruit fly GFRL
**Human GFRα1**	100%	61.5%	68.8%	9.9%
**Zebrafish GFRα1**	61.5%	100%	59.2%	10.3%
**Frog GFRα1**	68.8%	59.2%	100%	4.9%
**Fruit fly GFRL**	9.9%	10.3%	4.9%	100%

**Table 3:** Sequence identity comparisons of the amino acid sequences of human, zebrafish, frog and fruit fly RET.

	Human RET	Zebrafish RET	Frog RET	Fruit fly RET
**Human RET**	100%	57.9%	57.6%	25.5%
**Zebrafish RET**	57.9%	100%	57.9%	25.2%
**Frog RET**	57.6%	57.9%	100%	23.3%
**Fruit fly RET**	25.5%	25.2%	23.3%	100%

We investigated the crystal structure of the mammalian GDNF/GFRα1 complex to see if the contact surface in the mammalian GDNF/GFRα1 complex is conserved in zebrafish and frog. Of the fourteen listed contact points [18], only four were dissimilar in zGDNF and/or frog GDNF, and there was just one significant difference: mammalian Ile64 is retained in zGDNF but replaced with Lys in frog GDNF. As the contact residue in mammalian GFRα1 is Leu163, it is clear why zGDNF and hGDNF bind hGFRα1, while frog GDNF does not. (The other three changes are much less significant: human Leu111 is Ile in zGDNF and Leu in frog; human Ile122, Arg124 are replaced by Thr and Lys in both zebrafish and frog, with an equivalent change in GFRα1, from human Thr176 to Ser.)

**Fig 8:**
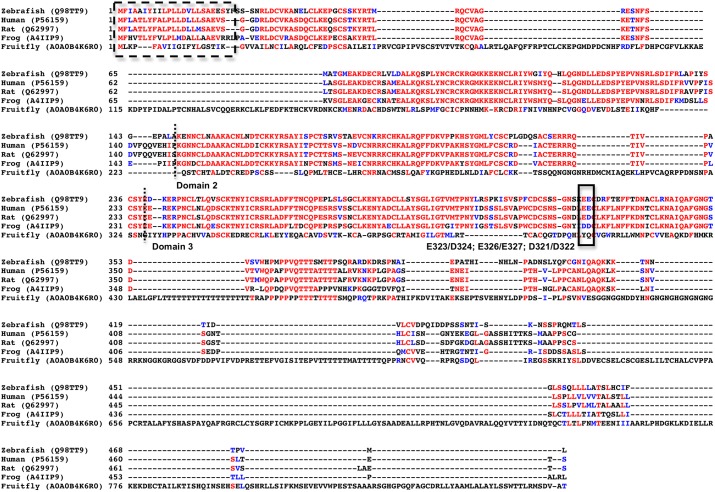
Amino acid sequence alignment of zebrafish, human, rat, frog and fruit fly GFRα1. The red letters indicate sequence identity and blue letter sequence similarity. Dashed box indicates signal sequence. The dotted line indicates the start and end of domains 2 and 3. Solid box indicates the amino acid E323/D324 (rat), E326/E327 (zebrafish), D321/D322 (frog) predicted to be involved in hRET complex formation.

Unfortunately, the atomic binding surfaces of the GFRα1/GDNF complex to RET are still unknown, so these could not be compared. We compared the mutation studies done in predicted rat GFRα1 RET binding hotspots [18]. The double mutant E323A/D324A in rat, corresponding to E326/E327 in zebrafish is slightly different in frog (D321/D322) while the human amino acids are exactly the same as the zebrafish (E323/E324) (Fig 8).

## Discussion

### The baculovirus/insect cell expression system

Our results show that insect cells can express correctly folded and fully functional components of the zebrafish RET signaling system. Little is known about the state of each protein once the complex forms, but our observation that zGDNF exists in both monomeric and dimeric forms is consistent with earlier data on GDNF [33, 34]. zRET in our hands was also produced in insect cells in both dimeric and monomeric form. This differs from previous studies of zRET [34], but is consistent with studies of human RET, which indicated that hRET exists in both monomer and dimer forms [35]. Since zRET^ecd^ (lacking the transmembrane and intracellular domain) is not in the membrane, we can only speculate what the true equilibrium state of the protein is. However, zRET^ecd^ did bind to the zGFRα1 / zGDNF complex with micromolar affinity (Fig 4*B*), consistent with previous measurements of the affinities of RET for the GFL/GFRα complex [11]. zRET^ecd^ is thus produced and correctly folded by the BEVS.

We also found that the functionality of zGDNF is sensitive to the position of the purification tag as well as the expression system used. Consistent with earlier experiments, placing a His-tag at the N-terminus was detrimental to the biological activity of GDNF [36]: there was significant reduction in the ability of His-tagged zGDNF to elicit RET-induced MAPK signaling (S2*A* Fig). The activity of zGFRα1 is not affected by tag placement, suggesting that neither the N- or C-terminal of GFRα1 interact with RET. Human GDNF produced in *E*. *coli* was also less functional in the phosphorylation and MAPK signaling experiments compared to zGDNF and the other controls (Figs 5 and 6*A*). We assume that this is either due to the lack of any post-translational modifications of the protein or a lower percentage of correctly folded protein from the *E*. *coli* expression system. Indeed, the unsuccessful clinical trials investigating the use of GDNF to treat Parkinson’s disease patients used “r-metHuGDNF”, which is a recombinant protein expressed in *E*. *coli* [2, 37]. Our results emphasize the importance of carefully considering tag placement when expressing ligands and also to thoroughly investigate the functionality of recombinant proteins used in clinical trials.

### *Drosophila* RET is not a good model for human RET

As expected, the fruit fly co-receptor did not activate the hRET signaling system: no GDNF orthologs have yet been reported for the fruit fly. Previous experiments done with dRET also report that hGDNF and hGFRα1 are not able to bind dRET^ecd^ [23] nor is dRET^ecd^ able to transduce GDNF mediated signaling, and here we confirm that dGFRL does not bind to hRET, or stimulate hRET signaling. This is most likely due to the sequence divergence and presumable structural divergence seen from the poorly conserved sequence identity (Tables 2 and 3) and from the alignments (Fig 8). There might not be a conserved RET/GFRα/GDNF signaling system in fruit fly and perhaps dRET might be constitutively held inactive by dGFRLs, as a part of a sorting receptor protein complex as has been suggested for other GFRα molecules [38]. These results question the role of dRET with a functional active tyrosine kinase domain as shown by Abrescia *et al*. [23]. A recent study provided evidence that fruit fly RET interacts with integrins *Mew* and *Myc* and together are essential for the regulation dendrite adhesion via *Rac1* signaling. They also question the existence of an unidentified RET ligand [39]. This suggests that there may still be a lot to be discovered about the roles of RET.

### Zebrafish GDNF and GFRα1 elicit signaling in human RET and promote the survival of mammalian neurons

Our key results show that baculovirus-produced non-mammalian GDNF and GFRα1 stimulate human RET phosphorylation as well as signal through the MAPK/ERK cascade to promote the survival of dopaminergic neurons affected by Parkinson’s disease. Our results suggest that there may have been more TH^+^ neurons in the samples treated with a lower concentration of zGDNF than in samples treated with a higher concentration (Fig 7B). There are three possible explanations for this result. First, lower neuronal survival at higher concentrations of zGDNF could be due to the fact that, while GDNF is known to stimulate TH [40], the reaction catalyzed by tyrosine hydroxylase generates reactive oxygen species [40], which in turn harm the cells, and so excessive levels of stimulation may lead to necrosis or apoptosis. Second, high GDNF concentrations may activate tyrosine phosphatases, thus decreasing net phosphorylation by RET. Third, as was shown for artemin and GFRα3 [11], a high concentration of zGDNF may shift the equilibrium from the heterotrimeric GFRα1_2_:GDNF that activates RET to the heterodimer GFRα1:GDNF.

While the fruit fly RET system by no means resembles the mammalian RET signaling and is not a good model system, the ability of zebrafish proteins to activate human proteins indicate such a degree of conservation that we can assume the atomic structure and especially the atomic binding surfaces are very similar. Also, while sequence identities of the frog RET protein orthologs were higher than those of the zebrafish, one difference (at least) in one essential binding residue (*i*.*e*. E326) in GFRα1 might make a difference. Our previous results have shown that mutations at this residue in GFRα1 significantly affected RET phosphorylation [18]. Our work thus suggests that an atomic resolution structure of the zebrafish RET complex structure [34], when it appears, will reveal the details of human RET interfaces, despite earlier claims to the contrary [21].

## Supporting information

S1 Fig zGDNF activates hRET phosphorylation as seen from ELISA assays. Luminescence readouts of ELISA assay of anti-phosphotyrosine detecting phosphorylated RET treated with various concentrations of zGDNF (black bars), hGDNF^BV^ (green bars) and hGDNF^IC^ (red bars) as positive controls, and media as a negative control; N = 1 experiment, with 3 repeats per experiment; statistically different from control **p<0.0001, *p<0.02. Error bars represent SEM.
(TIF)

S2 Fig The N-terminal 8x histidine tag negatively affects the ability of zGDNF to activate hRET signaling but does not affect zGFRα1. (A) luciferase activity readouts comparing different concentrations of His-tagged zGDNF, zGDNF, and hGDNF^Ec^ as a positive control. (B) luciferase activity readouts comparing the activity of His-zGFRα1 versus zGFRα1 with no tags using various concentrations of zGDNF. The dashed line indicates cleaved zGFRα1 and the solid line indicates His-tagged zGFRα1. N = 4 repeats per condition.
(TIF)

S3 Fig Human and zebrafish GDNF and GFRα1 do not activate MAPK/ERK signaling in the absence of hRET in the TrkB receptor cell-line. (A) zGDNF alone and (B) with zGFRα1 luciferase assay readouts with BDNF as a positive control. BDNF showed significant TrkB receptor activation with both 0.27 pM and 2.7 pM. N = 4 repeats per condition. Statistically different from control **p = 0.01; **p<0.0001. Error bars represent SEM.
(TIF)

S4 Fig zGDNF supports the survival of cultured murine sympathetic neurons. The percentage of survival of superior cervical ganglion neurons in the presence of zGDNF (black bars) compared to hGDNF^Ec^ (yellow bar) versus no GFL after 5 days; statistically different from control *p = 0.01; **p<0.0001. Error bars represent SEM.
(TIF)

S5 Fig Modeling the 3D mammalian GFRα1 structure against dGFRL. The protein sequence of dGFRL (residues 222–425; Uniprot: A0A0B4K6R0_DROME) was submitted to Phyre2 server [41] for homologous modeling. The best model (red) was selected (100% confidence) and compared to X-Ray structure of rat GFRα1 (green) in complex with human GDNF (cyan, PDBID: 3FUB) by superimposing the dGFRL with human GDNF. The resulted RMSD was 3.94Å including all atoms in the range (1048 atoms from each chain), or only 0.32Å if only secondary structure elements were included in the superimposition (731 atoms in each chain), when all the loops were excluded from comparison.
(TIF)
