# Supplementary figures and images for: Zebrafish GDNF and its co-receptor GFRα1 activate the human RET receptor and promote the survival of dopaminergic neurons in vitro
Source: PLoS One. 2017 May 3;12(5):e0176166. doi: 10.1371/journal.pone.0176166 (PMC5415192; doi:10.1371/journal.pone.0176166)

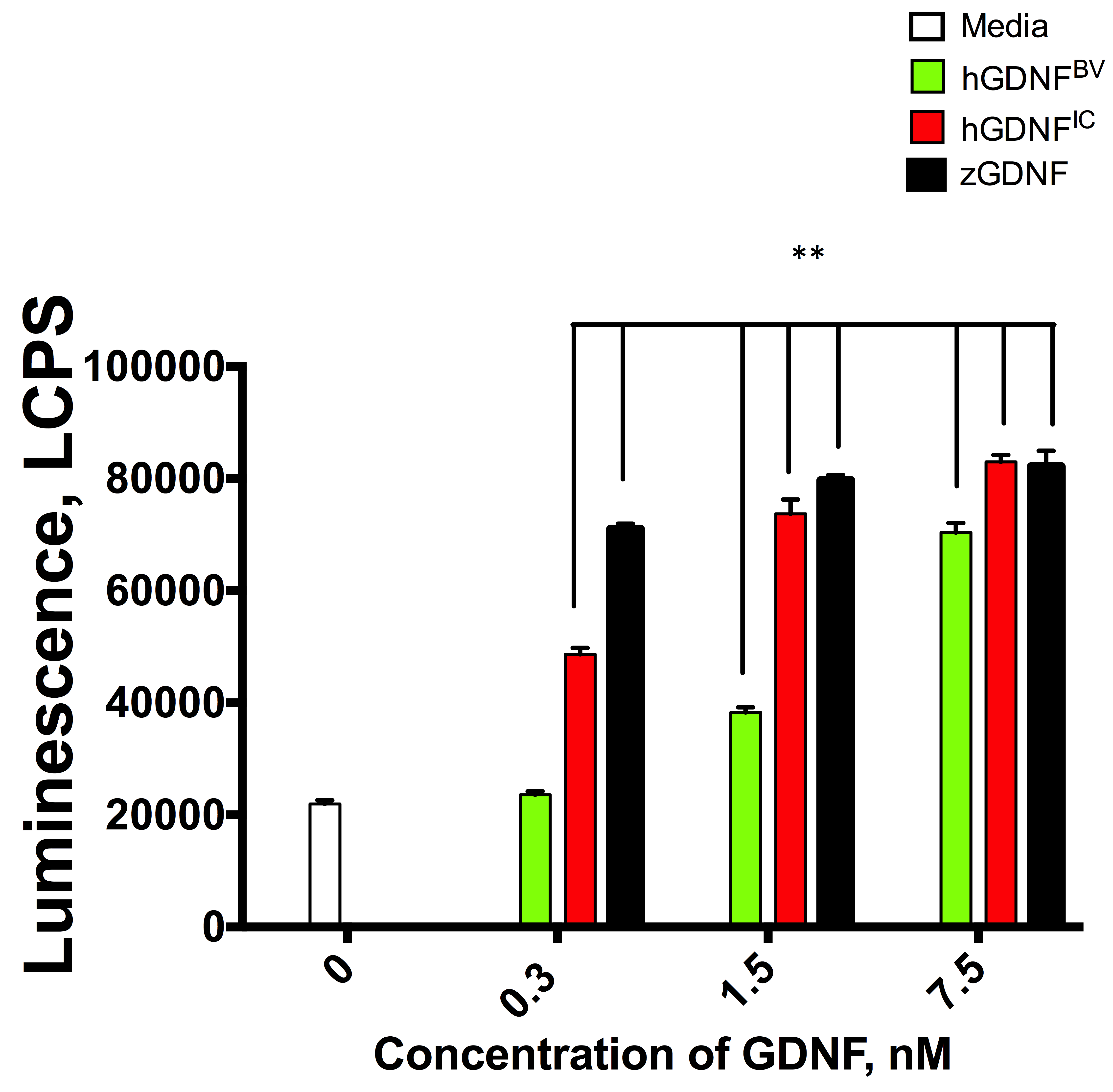

Supplement: S1 Fig — Luminescence readouts of ELISA assay of anti-phosphotyrosine detecting phosphorylated RET treated with various concentrations of zGDNF (black bars), hGDNFBV (green bars) and hGDNFIC (red bars) as positive controls, and media as a negative control; N = 1 experiment, with 3 repeats per experiment; statistically different from control **p<0.0001, *p<0.02. Error bars represent SEM. (TIF) [file pone.0176166.s001.tif]

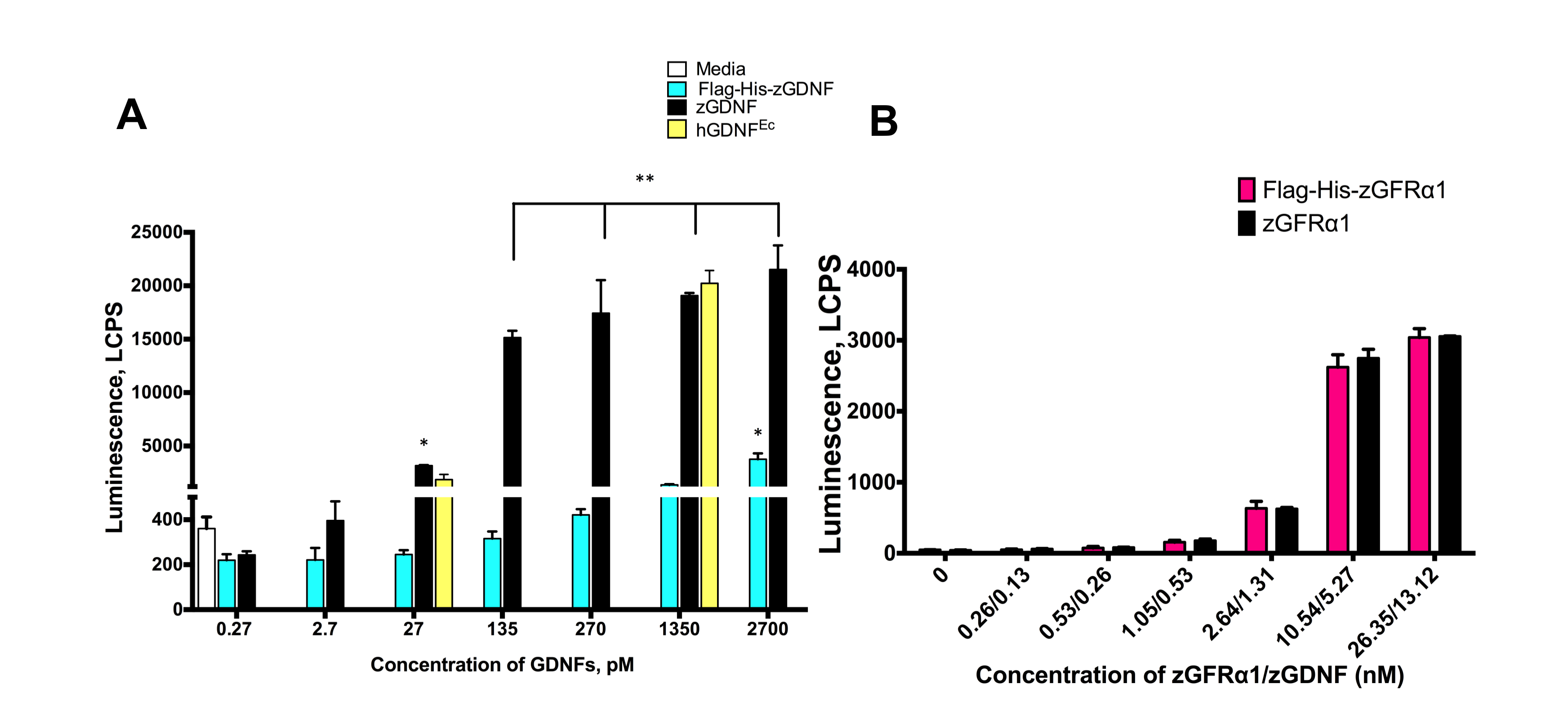

Supplement: S2 Fig — (A) luciferase activity readouts comparing different concentrations of His-tagged zGDNF, zGDNF, and hGDNFEc as a positive control. (B) luciferase activity readouts comparing the activity of His-zGFRα1 versus zGFRα1 with no tags using various concentrations of zGDNF. The dashed line indicates cleaved zGFRα1 and the solid line indicates His-tagged zGFRα1. N = 4 repeats per condition. (TIF) [file pone.0176166.s002.tif]

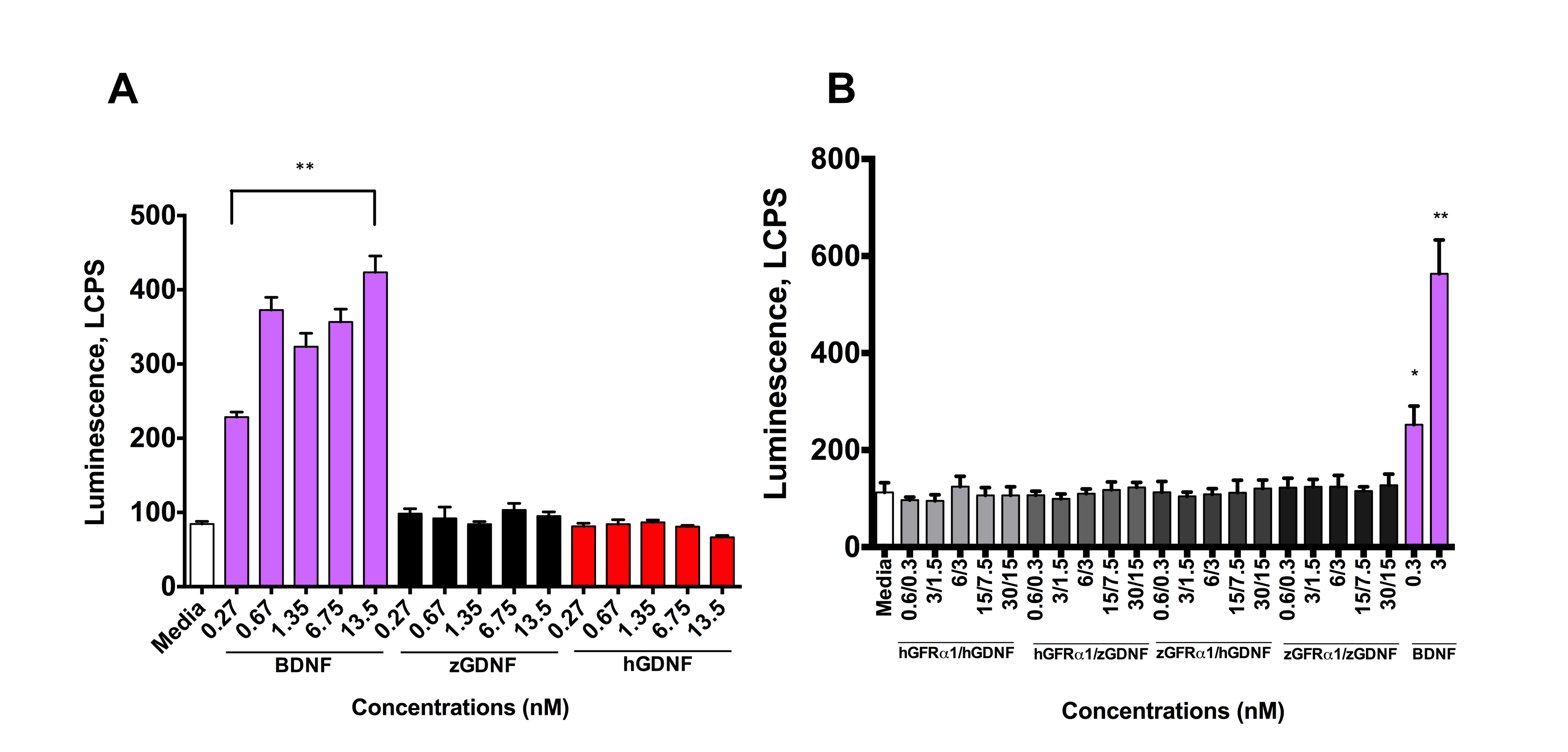

Supplement: S3 Fig — (A) zGDNF alone and (B) with zGFRα1 luciferase assay readouts with BDNF as a positive control. BDNF showed significant TrkB receptor activation with both 0.27 pM and 2.7 pM. N = 4 repeats per condition. Statistically different from control **p = 0.01; **p<0.0001. Error bars represent SEM. (TIF) [file pone.0176166.s003.tif]

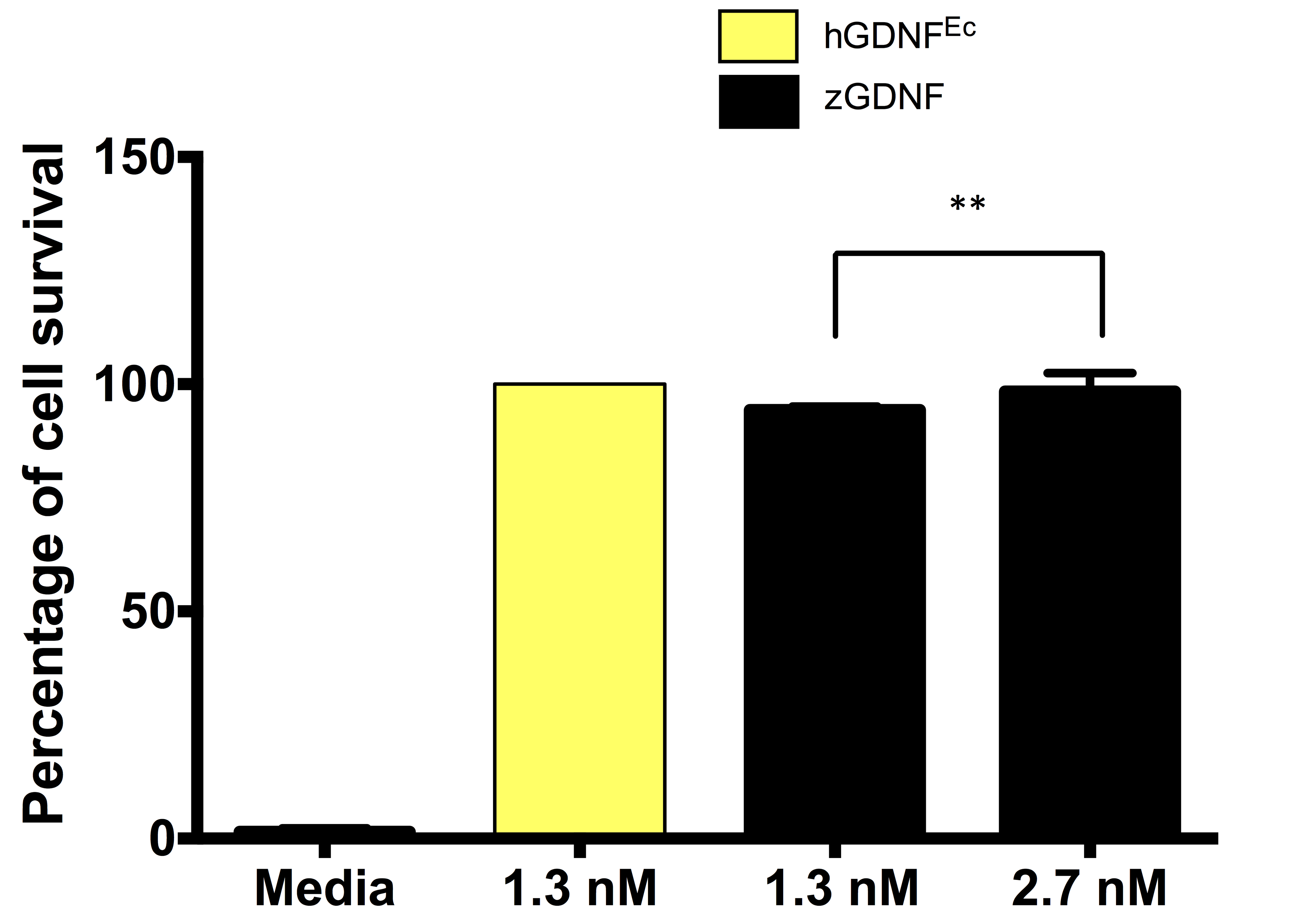

Supplement: S4 Fig — The percentage of survival of superior cervical ganglion neurons in the presence of zGDNF (black bars) compared to hGDNFEc (yellow bar) versus no GFL after 5 days; statistically different from control *p = 0.01; **p<0.0001. Error bars represent SEM. (TIF) [file pone.0176166.s004.tif]

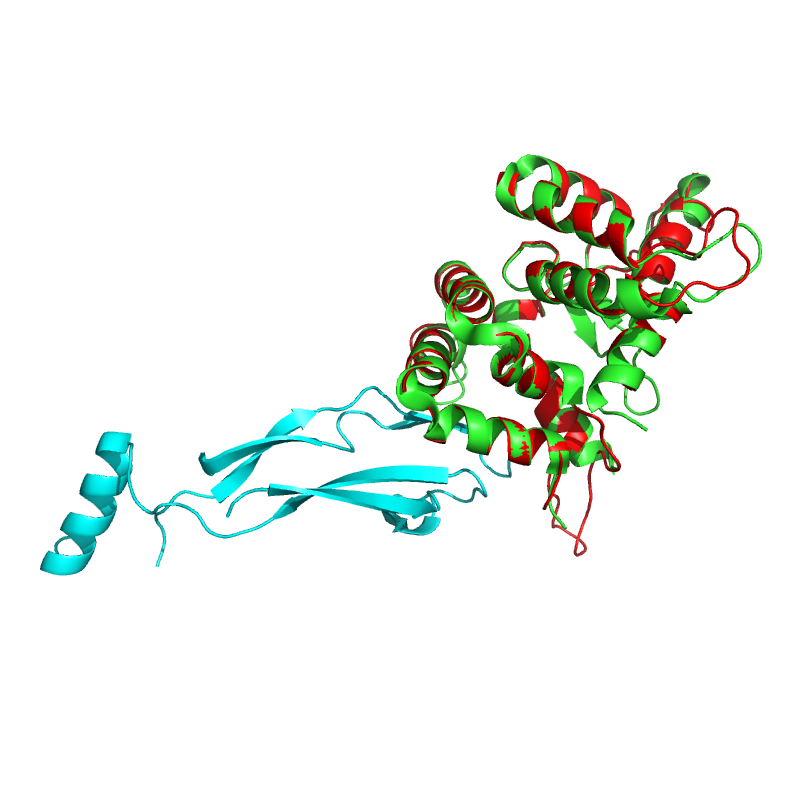

Supplement: S5 Fig — The protein sequence of dGFRL (residues 222–425; Uniprot: A0A0B4K6R0_DROME) was submitted to Phyre2 server [41] for homologous modeling. The best model (red) was selected (100% confidence) and compared to X-Ray structure of rat GFRα1 (green) in complex with human GDNF (cyan, PDBID: 3FUB) by superimposing the dGFRL with human GDNF. The resulted RMSD was 3.94Å including all atoms in the range (1048 atoms from each chain), or only 0.32Å if only secondary structure elements were included in the superimposition (731 atoms in each chain), when all the loops were excluded from comparison. (TIF) [file pone.0176166.s005.tif]
